# Hypermethylation of the TSPOAP1-AS1 Promoter May Be Associated with Obesity in Overweight/Obese Korean Subjects

**DOI:** 10.3390/ijms21093307

**Published:** 2020-05-07

**Authors:** Nam-Hui Yim, Min Ho Cha, Myung Sunny Kim

**Affiliations:** 1 Korean Medicine (KM) Application Center, Korea Institute of Oriental Medicine (KIOM), 70 Cheomdan-ro, Dong-gu, Daegu 41062, Korea; nhyim23@kiom.re.kr; 2 Healthcare Research Group, Korea Food Research Institute, Wanju 55365, Korea; truka@kfri.re.kr; 3 Department of Food Biotechnology, Korea University of Science & Technology, Wanju 55365, Korea

**Keywords:** TSPOAP1-AS1, obesity, DNA methylation, body mass index, total cholesterol, LDL-cholesterol

## Abstract

Obesity is a major chronic disease associated with the risk of serious cardiovascular or endocrinal diseases, such as hypertension, diabetes, atherosclerosis and stroke. Considerable interest has been directed towards the potential effects of epigenetic variations in obesity. In this study, we evaluated DNA methylation level at the promoter region of the gene encoding TSPO-associated protein 1 antisense RNA 1 (TSPOAP1-AS1) in 80 overweight/obese subjects (body mass index (BMI) > 25) and 104 non-obese subjects who participated in the SOPI-Stroke study in Korea. DNA methylation was measured using bisulfite amplicon sequencing (BSAS). A general linear model or relative correlation was used to determine the effects of DNA methylation on obesity and obese phenotypes. Notably, the mean level of DNA methylation was significantly higher in the overweight/obese group than in the non-obese group (18.62% vs. 17.18%). Further analyses revealed significant positive correlations of the BMI, the serum total cholesterol and low-density lipoprotein cholesterol levels with the DNA methylation level (*p* = 0.0493, *p* = 0.003, and *p* = 0.0094, respectively). The study findings suggest an association between DNA methylation at the *TSPOAP1-AS1* promoter and overweight/obesity. Accordingly, methylation in this promoter region might be a potential predictor of obesity.

## 1. Introduction

Obesity is one of major epidemic diseases associated with increased risk of other cardiovascular and metabolic diseases, such as type 2 diabetes, coronary heart disease, stroke and cancer. The prevalence of obesity is continuing to increase worldwide, affecting approximately 93.3 million adults in the USA, and this continuously increasing trend is displayed in Asia [1,2].

Many studies of the underlying causes of obesity have identified potential associations with epigenetic variants [3,4,5]. Particularly, studies have persistently identified the effects of genetic variation induced by changes in DNA methylation in the genomes of obese humans [6,7]. These epigenetic variations associated with obesity, which affect the activity and expression of the targeted genes, have been attributed to environmental factors, including diet, weight gain/loss, smoking, and alcohol consumption [8]. Consequent changes in the methylation status of target gene promoters could therefore be considered putative markers of obesity.

Translocator protein (TSPO)-associated protein 1 antisense RNA 1 (TSPOAP1-AS1; also benzodiazepine-associated protein 1 antisense RNA 1/BZRAP1-AS1) is a long non-coding RNA (lncRNA) encoded on chromosome 17. This lncRNA regulates the expression of peripheral-type *TSPOAP1*, which encodes a benzodiazepine receptor-associated mitochondrial protein associated with the neurotransmitter release cycle and benzodiazepine pathway [9]. Both TSPOAP1 and TSPOAP1-AS1 were shown to be correlated with Alzheimer’s disease (AD) progression through interactions with a risk factor gene of AD, trophoblast glycoprotein (TPBG) [10,11]. Recently, TSPOAP1-AS1 has also been identified as a potential biomarker of malignancies, including breast and prostate cancers [12]. Until now, however, the function and possible role of TSPOAP1-AS1 in diseases such as obesity remain unknown, but some studies suggested the possibility of relation between TSPOAP1-AS1 and obesity Blasi showed that the agonist of benzodiazepines receptor (translocator protein: TSPO) reduced body weight gain and lowered insulin level in obese rat [13]. Other studies reported that expression is highly reduced in adipocyte and macrophage of adipose tissue in obese mouse [14]. TSPOAP1 specifically interacts with TSPO [15]. These finding mean that TSPOAP1 and TSPOAP1-AS1 might affects obesity. 

In this study, we investigated the association between epigenetic alterations and obesity by elucidating the DNA methylation levels at CpG islands in the *TSPOAP1-AS1* promoter region in normal and overweight/obese Korean subjects.

## 2. Results

### 2.1. Characteristics of Subjects

Table 1 shows the general characteristics between normal and overweight/obese subjects. Total 184 subjects comprising 104 of normal and 80 of overweight/obese were enrolled in this study. Obese phenotypes, such as BMI, waist circumferenc, and waist/hip ratio (WHR), were significantly high in overweight/obese subjects comparing to those of normal subjects (BMI, *p* < 0.001; waist circumference, *p* < 0.001; WHR, *p* = 0.002). Additionally, among blood parameters, GPT and triglyceride in overweight/obese subjects were significantly higher than those in normal subjects (GPT, *p* = 0.003; triglyceride, *p* = 0.011). 

### 2.2. GpG Island in TSPOAP1-AS1 Promoter Region

**Figure 1:**
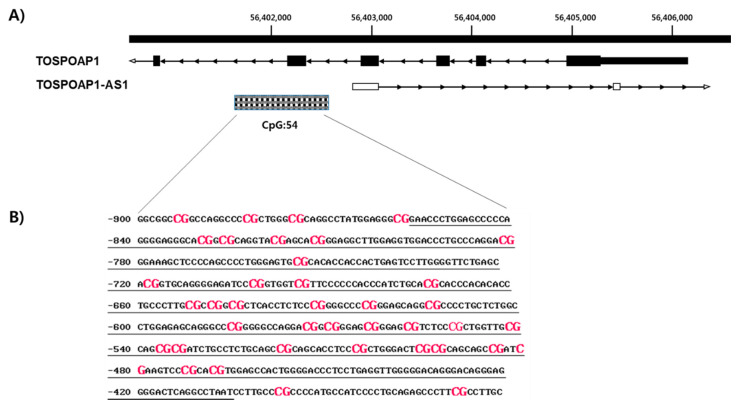
Analysis of hypermethylated CpG sites in TSPOAP1-AS1 promoter region. (**A**)Black box and arrow line showed the TSPOAP1-AS1 and TSPOAP1 gene and dot box indicate CpG island region located at 5′-flanking region of TSPOAP1-AS1. Dot line means the position of DNA methylation analysis in this study. (**B**) The sequence of TSPOAP1-AS1 promoter region. The CpG site was shown in red label and the analyzed region in this study showed in underline.

*TSPOAP1-AS1* located at positive strand of chromosome 17. Using a genomic map of *TSPOAP1-AS1* (NR_038410), we confirmed a CpG island in the 5′-upstream region (CGI: 54; Figure 1A) that contained 33 CpG sites (Figure 1B). Previous pilot study of whole genome bisulfate sequencing among each of four normal subjects and overweight/obese subjects, we found hypermethylation of CGI:54 on overweight/obese subjects (Table S1). Based on this data, we analyzed DNA methylation level at each CpG site in the *TSPOAP1-AS1* promoter region.

### 2.3. Comparison of TSPOAP1-AS1 Methylation between Normal and Overweight/Obese Subjects

**Figure 2:**
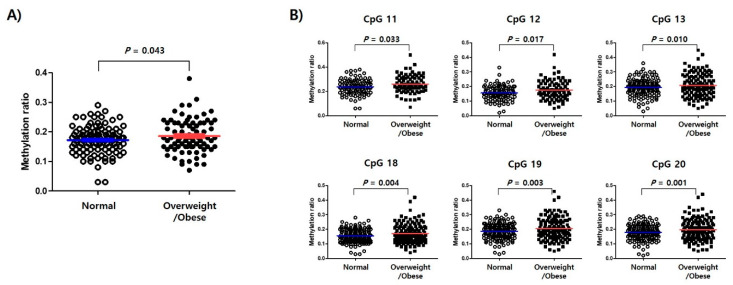
TSPOAP1-AS1 methylation associated with obesity. (**A**) Distribution of mean DNA methylation level on normal and overweight/obese subjects. (**B**) Significance of hypermethylation at the specific CpG sites of TSPOAP1-AS1 promoter region in overweight/obese subjects compared to normal subjects.

Normal and overweight/obese subjects (Table S2) had mean DNA methylation levels of 17.18 ± 4.65% and 18.62 ± 5.71%, respectively, at these 33 CpG sites (Figure 2A). Particularly, overweight/obese subjects had significantly higher methylation levels at the sites CpG11 (*p* = 0.033), CpG12 (*p* = 0.017), CpG13 (*p* = 0.010), CpG18 (*p* = 0.004), CpG19 (*p* = 0.003), and CpG20 (*p* = 0.001; Figure 2B). 

### 2.4. Comparison of TSPOAP1-AS1 Methylation Associated with Obesity-Related Phenotypes between Normal and Overweight/Obese Subjects

**Figure 3:**
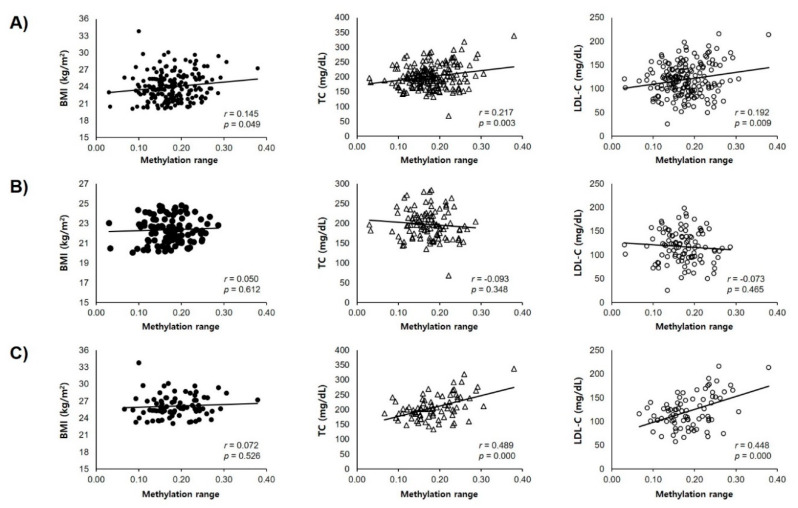
Analysis of the correlations between DNA methylation of the *TSPOAP1-AS1* promoter region and BMI, Total cholesterol (TC), and LDL-cholesterol (LDL-C). (**A**) Total of normal and overweight/obese subjects; (**B**) Normal subjects; (**C**) Overweight/obese subjects.

A further analysis of the correlations between DNA methylation of the *TSPOAP1-AS1* promoter region and obesity-related markers yielded a significantly positive correlation with BMI (*r* = 0.1451, *p* = 0.0493; Figure 3A) and positive but non-significant links with waist circumference and the waist-to-hip ratio (data not shown). An analysis of correlations between DNA methylation and blood parameters revealed significant associations with the levels of serum total cholesterol (TC, *r* = 0.2174, *p* = 0.003) and low-density lipoprotein -cholesterol (LDL-C, *r* = 0.1916, *p* = 0.0094; Figure 3A). Especially, the methylation level showed significant correlation with TC (*r* = 0.489, *p* < 0.001) and LDL-C (*r* = 0.488, *p* < 0.001) in overweight/obese subjects, but not in normal (Figure 3B,C).

Table 2 presents the correlations of methylation at each CpG locus with BMI, the serum total cholesterol and LDL-cholesterol levels. Notably, the methylation levels at six CpG sites were strongly associated with both the serum total cholesterol and LDL-cholesterol levels.

### 2.5. Prediction of the Binding Affinity with Transcription Factors at the Specific CpG Sites in TSPOAP1-AS1 Promoter Region

**Figure 4:**
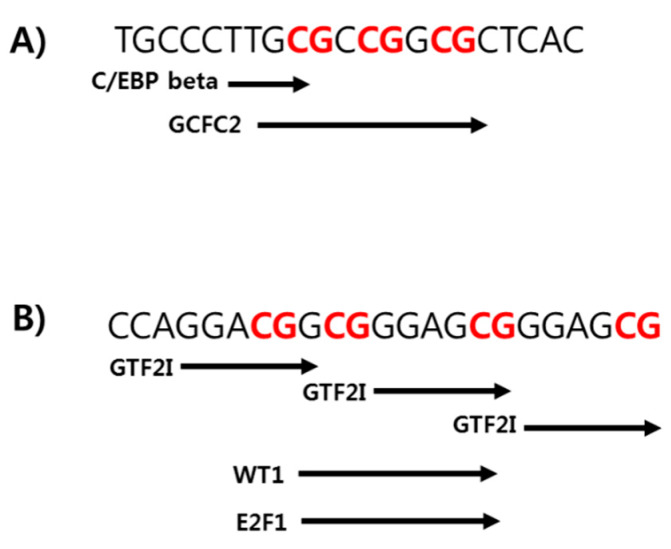
Prediction binding site analysis of *TSPOAP1-AS1* promoter region. PROMO/transcription factor binding sites (TFBS) analysis on the sequences of CpG sites 11–13 (**A**) and 18–20 (**B**) of the *TSPOAP1-AS1* promoter region. Each black arrow indicates the specific transcription factor identified its predicted binding sequence.

As a change in DNA methylation at only one CpG site can affect the binding affinity of a transcription factor for the targeted DNA sequence, thus affecting gene expression, we used PROMO to perform the TFBS analysis. Specifically, we aimed to identify specific transcription factors for which binding to the target DNA might be affected by methylation at CpG sites 11–13 and 18–20 in the *TSPOAP1-AS1* promoter region. This analysis revealed that CCAAT/enhancer-binding protein (C/EBP) β and Wilms tumor protein-1 (WT-1) might be affected by methylation at CpG sites 11 and 19–20, respectively (Figure 4).

## 3. Discussion

Obesity is a serious social disease many countries have aimed to prevent and decrease the occurrence [2]. This epidemic has inspired various attempts to identify causal genetic/epigenetic factors at multiple adiposity-associated, differentially methylated sites using epigenome-wide association approaches (e.g., genome-wide quantification of site-specific DNA methylation) [16,17,18]. Epigenetic changes, particularly changes in methylation at specific target gene loci, are expected to be correlated with and predictive of obesity [19,20,21]. Several recent studies have also suggested correlations of different methylation patterns with various obesity-associated traits, such as BMI, wait circumference (WC), and blood lipid parameters [22,23]. For example, a strong correlation has been reported between the hypoxia-inducible factor 3-alpha gene (*HIF3A*) and BMI, and methylation of this gene is associated with obesity and adipose tissue dysfunction [24]. Notably, Richmond et al. identified an association of BMI and novel methylation sites of *HIF3A*, indicating that this epigenetic factor might be related to adiposity.

In this study, we demonstrated an association of hypermethylation in the *TSPOAP1-AS1* promoter region with overweight/obesity in a sample of Korean subjects. Specifically, we detected associations of methylation at different CpG sites with the incidence of overweight/obesity (Figure 2), as well with the BMI and lipid parameters (TC and LDL-cholesterol; Figure 3). These results support the hypothesis that the methylation of *TSPOAP1-AS1* might be associated with obesity and suggest a possible application for this epigenetic factor as a predictor of health outcome.

TSPOAP1-AS1 binds to and regulates TSPO, an outer-mitochondrial membrane translocator that participates in mitochondrial permeability, reactive oxygen species (ROS) regulation, cellular energy production and cholesterol uptake [25,26]. In a previous study, obese mice exhibited a significant decrease in adipose tissue-specific TSPO expression, suggesting a potential role for TSPO/TSPOAP1 in adipose tissue homeostasis [14]. Accordingly, we hypothesized that TSPOAP1-AS1 might also contribute to adipose tissue homeostasis by regulating TSPOAP1 protein synthesis. Other studies have focused mainly on TSPOAP1-AS1 as a modulating factor in cancers and potential biomarker or onco-target. For example, Giulietti et al. investigated TSPOAP1-AS1 as a new potential diagnostic/prognostic biomarker of pancreatic ductal adenocarcinoma through a weighted gene co-expression work analysis (WGCNA) [27]. An association of methylation of *TSPOAP1-AS1* with prostate cancer was identified previously [28], and TSPOAP1-AS1 was found to associate with metastasis and cancer recurrence in patients with breast cancer [12]. Regarding non-malignant diseases, a recent study by Witoelar et al. revealed an association of TSPOAP1-AS1 with AD [10]. In that study, a genome wide association study (GWAS) of genetic risk factors for AD identified four genomic loci, including TSPOAP1-AS1. Notably, TSPOAP1-AS1 was found to be associated significantly with AD via interactions with APOEε4 allele, a major risk factor for late-onset AD [11].

In this study, we identified specific methylated CpG sites related to epigenetic phenotypes in TSPOAP-AS1. We hypothesized that the phenotypic features and serum cholesterol levels associated with obesity might be correlated strongly with the methylation of selected CpG sites in the *TSPOAP1-AS1* promoter region. Consistent with this hypothesis, our *TSPOAP1-AS1* binding site prediction data suggested that CpG sites within the ranges of 11–13 and 18–20 would be bound by C/EBP β and WT-1, respectively. C/EBP β contributes to adipocyte differentiation by inducing the expression of two master adipogenic transcription factors, C/EBP α and peroxisome proliferator-activated receptor (PPAR) γ [29,30]. According to Kowenz-Leutz et al., the phosphorylation of C/EBP β leads to arginine methylation, which interferes with epigenetic processes during adipogenesis [31]. WT-1 plays multiple roles in the development and homeostasis of various tissues and organs [32,33,34]. In a previous study that compared the DNA methylation patterns of high and low responders to a hypocaloric diet, the former group exhibited higher levels of methylation at several CpG sites in the *WT1* promoter [35]. Furthermore, the weight loss intervention for obese stroke patients induced changes in the methylation patterns on *WT1* [36]. Taken together, our results suggest a potential role for TSPOAP1-AS1 in the epigenetic modifications of obesity-related genes. In other words, TSPOAP1-AS1 may be a useful epigenetic biomarker of obesity.

This study had some limitations of note: the small sample size makes it difficult to generalize the results, and the age of enrolled subjects is old. Generally, epigenetic variations are age-dependent, an association that has been clearly demonstrated in age-dependent diseases [8]. Nevertheless, the increasing prevalence of obesity in middle-aged populations underscores the need for additional studies of this age group. Third, this study did not explore the functional effect of hypermethylation at the *TSPOAP1-AS1* promoter. Despite these limitations, however, this study is the first to explore the association between *TSPOAP1-AS1* promoter methylation and obesity. Further studies with large populations should be conducted to confirm and generalize our conclusions.

## 4. Methods

### 4.1. Study Participants

This study was conducted as part of “The Fundamental Study for the Standardization and Objectification of PI in TKM for Stroke (SOPI-Stroke)”, a project of the Korean Institute of Oriental Medicine (KIOM) [37]. The study was approved by the Institutional Review Board (IRB No. I-1603/001-001-01) of the KIOM. Clinical data were collected after obtaining written informed consent from all subjects.

The study included overweight/obese subjects who were admitted between 2005 and 2010 to the following participating Korean hospitals: Korean Medicine Hospital of Daejeon University (Daejeon, Korea) and Korean Medicine Hospital of Wonkwang University (Iksan, Korea). Overweight or obesity was defined as a body mass index (BMI) > 25 kg/m^2^ or BMI > 30 kg/m^2^ according to the World Health Organization (WHO) guideline [38]. Subjects with a BMI < 25 kg/m^2^ were also included as the normal weight group. Patients who had been diagnosed previously with hypertension, diabetes, hyperlipidemia, coronary heart disease and stroke were excluded from this study.

### 4.2. DNA Methylation Analysis

DNA methylation of the TSPOAP1-AS1 promoter region was analyzed using the bisulfite amplicon sequencing (BSAS) method, which was performed by the Life is Art of Science (LAS) Laboratory (Gimpo-si, Korea). Briefly, genomic DNA was prepared from a blood sample of each subject using the Gene All genomic DNA extraction kit (GeneAll, Seoul, Korea). The DNA was subsequently bisulfite-converted using EZ DNA Methylation according to the manufacturer’s protocol (Zymo Research, Irvine, CA, USA). The following PCR primers were used to amplify the *TSPOAP1-AS1* promoter region: gene-F, 5′-GGAATTTTGGAGTTTTTAGGGG-3′ and gene-R, 5′-ATTAAACCTAAATCCCCTCCCTATC-3′. A PCR library was generated from the amplicons using an Illumina TruSeq Nano DNA sample prep kit (Illumina, San Diego, CA, USA) according to the manufacturer′s instructions. The prepared library was sequenced on a Miseq system (Illumina, Inc., San Diego, CA, USA) with 300-bp paired-end reads. A map and methylation level profile of each CpG position in the *TSPOAP1-AS1* promoter region were generated using BS-seeker2 according to the UCSC hg19 reference sequence [39].

### 4.3. Prediction of Transcription Factor Binding

To detect potential the regulatory effects of methylation at the investigated CpG sites, we searched for TSPOAP1-AS1 putative transcription factor binding sites (TFBS) in the polymorphic site using PROMO, a freely available online tool [40]. Predictions were made after limiting the search to the human species and transcription factors and setting the minimum sequence similarity threshold for TF binding detection to 85% as suggested by the TRANSFAC database (v.8.3) utilized by PROMO.

### 4.4. Statistical Analysis

SPSS19.0 software (SPSS, Seoul, Korea) was used to perform the statistical analysis. The chi-square test was used to compare differences between categorical variables. Normality of continuous variables was decided by Kolmogorov-Smirnov test, and statistical difference was determined by Student’s t-test or binary general linear model adjusted smoking status and alcohol consumption status. The correlations of DNA methylation with overweight/obesity or blood parameters were measured using a two-way Pearson’s correlation. Statistical significance was determined as a *p* value < 0.05.

### 4.5. Availability of Data and Materials

All datasets used and analyzed during the current study are available from the corresponding author on reasonable request.

### 4.6. Ethics Approval and Consent to Participate

Patients who were willing to participate provided written informed consent. This study was approved by the Institutional Review Boards (IRB No. I-1603/001-001-01) of the KIOM.

## 5. Conclusions

In conclusion, our evaluation of DNA methylation of the *TSPOAP1-AS1* promoter region revealed an association of hypermethylation in this region with overweight/obese adult in Korea. We further identified associations of methylation at specific CpG sites in the promoter region of *TSPOAP1-AS1* with serum lipid levels. We note that TSPOAP1-AS1 was not previously explored in the context of obesity. Therefore, our initial finding TSPOAP1-AS1 methylation may be an epigenetic biomarker of obesity merits further research although recommending the additional studies to explore the relationship between *TSPOAP1-AS1* methylation and obesity. 

## Figures and Tables

**Table 1:** Characteristics of normal and overweight/obese subjects.

Characteristics	Normal	Obesity	*p*-Value
*n*	104	80	
*Anthropometric characteristics*			
Sex (M/F)	64/40 *	31/49	0.003 ^a^
Age (years)	59.84 ±10.68 ^#^	64.04 ±9.87	0.007 ^b^
Smoking (none/stop/active)	55/31/18	56/16/8	0.061
Alcohol consumption (none/former/active)	50/9/45	47/5/27	0.307
Weight (kg)	59.15 ± 7.84	65.16 ± 9.22	<0.001
BMI (kg/m^2^)	22.35 ± 1.33	26.12 ± 1.96	<0.001
Waist circumference (cm)	80.24 ± 6.93	89.02 ± 6.06	<0.001
WHR	0.869 ± 0.056	0.895 ± 0.046	0.002
*Medical history*			
Depression (Yes, %)	0 (0.0) *	5 (6.3)	0.013
Migraine (Yes, %)	9 (8.7)	12 (15.2)	0.241
*Blood parameters*			
WBC (×10^3^)	5.72 ± 1.49	5.82 ± 1.22	0.348 ^c^
RBC (×10^6^)	4.51 ± 0.45	4.46 ± 0.40	0.306
Hg (g/dL)	13.90 ± 1.45	13.75 ± 1.31	0.103
Hct (%)	41.54 ± 4.15	40.86 ± 3.73	0.223
Platelet (×10^3^/µL)	184.77 ± 75.92	208.01 ± 78.61	0.199
GOT (U/dL)	23.40 ± 6.96	25.84 ± 18.23	0.107
GPT (U/dL)	19.76 ± 8.45	25.19 ± 20.53	0.003
Total cholesterol (mg/dL)	197.59 ± 36.84	206.92 ± 40.90	0.285
Triglyceride (mg/dL)	125.63 ± 70.26	153.57 ± 75.21	0.011
HDL-cholesterol (mg/dL)	56.24 ± 12.80	55.29 ± 12.18	0.110
LDL-cholesterol (mg/dL)	117.53 ± 33.41	120.92 ± 34.65	0.772
FBS (mg/dL)	97.88 ± 9.76	97.95 ± 11.62	0.399

* Indicates the number of subjects (%). ^#^ Indicates the mean ± standard deviation. Categorical variables were analyzed using the chi-squared test or Fisher’s test ^b^, and continuous variables were analyzed using Student’s t-test ^a^ or a binary general linear model after adjusting sex, age, smoking and alcohol consumption ^c^. BMI: body mass index, WHR: waist/hip ratio, WBC: white blood cell, RBC: red blood Cell, Hg: hemoglobin, Hct: hematocrit, GOT: glutamic oxaloacetic transaminase, GPT: glutamic pyruvate transaminase.

**Table 2:** Association between specific methylation of the *TSPOAP1-AS1* promoter region and obesity-related markers.

CpG No.	Loci at Chromosome	BMI		Total Cholesterol		LDL-Cholesterol	
		*r*	*p*	*r*	*p*	*r*	*p*
11	56402159	0.138	0.062	0.205	0.005	0.188	0.011
12	56402162	0.183	0.013	0.244	0.001	0.197	0.008
13	56402165	0.186	0.012	0.226	0.002	0.193	0.009
18	56402238	0.180	0.015	0.266	<0.001	0.232	0.002
19	56402241	0.185	0.012	0.217	0.003	0.212	0.004
20	56402247	0.214	0.004	0.227	0.002	0.195	0.008

## Supplementary Materials

Supplementary materials can be found at https://www.mdpi.com/1422-0067/21/9/3307/s1.

## Abbreviations

TSPO: translocator protein 18K; TSPOAP1-AS1: TSPO-associated protein 1 antisense RNA 1; BZRAP1-AS1: benzodiazepine-associated protein 1 antisense RNA 1; lncRNA: long non-coding RNA; AD: Alzheimer’s disease; TPBG: trophoblast glycoprotein; SOPI-Stroke: Standardization and Objectification of PI in TKM for Stroke; BMI: body mass index; WHO: World Health Organization; BSAS: bisulfite amplicon sequencing; TFBS: transcription factor binding sites; WC: waist circumference; WHR: waist-to-hip ratio; TC: total cholesterol; LDL: low-density lipoprotein; HDL: high-density lipoprotein; C/EBP: CCAAT/enhancer-binding protein; WT-1: Wilms tumor protein-1; HIF3A: hypoxia-inducible factor 3-alpha; ROS: reactive oxygen species; WGCNA: weighted gene co-expression work analysis; GWAS: genome wide association study; PPAR: peroxisome proliferator-activated receptor

## References

[1] Dietz W.H. (2015). The response of the US Centers for Disease Control and Prevention to the obesity epidemic. Annu. Rev. Public Health.

[2] Ramachandran A., Snehalatha C. (2010). Rising burden of obesity in Asia. J. Obes..

[3] van Dijk S.J., Molloy P.L., Varinli H., Morrison J.L., Muhlhausler B.S., Members of Epi S. (2015). Epigenetics and human obesity. Int. J. Obes..

[4] Rao K.R., Lal N., Giridharan N.V. (2014). Genetic & epigenetic approach to human obesity. Indian J. Med. Res..

[5] Soubry A., Guo L., Huang Z., Hoyo C., Romanus S., Price T., Murphy S.K. (2016). Obesity-related DNA methylation at imprinted genes in human sperm: Results from the TIEGER study. Clin. Epigenet..

[6] Guay S.P., Brisson D., Lamarche B., Biron S., Lescelleur O., Biertho L., Marceau S., Vohl M.C., Gaudet D., Bouchard L. (2014). ADRB3 gene promoter DNA methylation in blood and visceral adipose tissue is associated with metabolic disturbances in men. Epigenomics.

[7] Garcia-Cardona M.C., Huang F., Garcia-Vivas J.M., Lopez-Camarillo C., Del Rio Navarro B.E., Navarro Olivos E., Hong-Chong E., Bolanos-Jimenez F., Marchat L.A. (2014). DNA methylation of leptin and adiponectin promoters in children is reduced by the combined presence of obesity and insulin resistance. Int. J. Obes..

[8] Brunet A., Berger S.L. (2014). Epigenetics of aging and aging-related disease. J. Gerontol. A Biol. Sci. Med. Sci..

[9] Mittelstaedt T., Schoch S. (2007). Structure and evolution of RIM-BP genes: Identification of a novel family member. Gene.

[10] Witoelar A., Rongve A., Almdahl I.S., Ulstein I.D., Engvig A., White L.R., Selbaek G., Stordal E., Andersen F., Braekhus A. (2018). Meta-analysis of Alzheimer’s disease on 9751 samples from Norway and IGAP study identifies four risk loci. Sci. Rep..

[11] Jun G.R., Chung J., Mez J., Barber R., Beecham G.W., Bennett D.A., Buxbaum J.D., Byrd G.S., Carrasquillo M.M., Crane P.K. (2017). Transethnic genome-wide scan identifies novel Alzheimer’s disease loci. Alzheimers Dement..

[12] Li J., Wang W., Xia P., Wan L., Zhang L., Yu L., Wang L., Chen X., Xiao Y., Xu C. (2018). Identification of a five-lncRNA signature for predicting the risk of tumor recurrence in patients with breast cancer. Int. J. Cancer.

[13] Blasi C. (2000). Influence of benzodiazepines on body weight and food intake in obese and lean Zucker rats. Prog. Neuropsychopharmacol. Biol. Psychiatry.

[14] Thompson M.M., Manning H.C., Ellacott K.L.J. (2013). Translocator Protein 18 kDa (TSPO) Is Regulated in White and Brown Adipose Tissue by Obesity. PLoS ONE.

[15] Galiegue S., Jbilo O., Combes T., Bribes E., Carayon P., Le Fur G., Casellas P. (1999). Cloning and characterization of PRAX-1. A new protein that specifically interacts with the peripheral benzodiazepine receptor. J. Biol. Chem..

[16] Wang X.L., Zhu H.D., Snieder H., Su S.Y., Munn D., Harshfield G., Maria B.L., Dong Y.B., Treiber F., Gutin B. (2010). Obesity related methylation changes in DNA of peripheral blood leukocytes. BMC Med..

[17] Xu X.J., Su S.Y., Barnes V.A., De Miguel C., Pollock J., Ownby D., Shi H.D., Zhu H.D., Snieder H., Wang X.L. (2013). A genome-wide methylation study on obesity Differential variability and differential methylation. Epigenetics.

[18] Demerath E.W., Guan W.H., Grove M.L., Aslibekyan S., Mendelson M., Zhou Y.H., Hedman A.K., Sandling J.K., Li L.A., Irvin M.R. (2015). Epigenome-wide association study (EWAS) of BMI, BMI change and waist circumference in African American adults identifies multiple replicated loci. Hum. Mol. Genet..

[19] Wahl S., Drong A., Lehne B., Loh M., Scott W.R., Kunze S., Tsai P.C., Ried J.S., Zhang W.H., Yang Y.W. (2017). Epigenome-wide association study of body mass index, and the adverse outcomes of adiposity. Nature.

[20] Dick K.J., Nelson C.P., Tsaprouni L., Sandling J.K., Aissi D., Wahl S., Meduri E., Morange P.E., Gagnon F., Grallert H. (2014). DNA methylation and body-mass index: A genome-wide analysis. Lancet.

[21] Shah S., Bonder M.J., Marioni R.E., Zhu Z.H., McRae A.F., Zhernakova A., Harris S.E., Liewald D., Henders A.K., Mendelson M.M. (2015). Improving Phenotypic Prediction by Combining Genetic and Epigenetic Associations. Am. J. Hum. Genet..

[22] Sayols-Baixeras S., Subirana I., Fernandez-Sanles A., Senti M., Lluis-Ganella C., Marrugat J., Elosua R. (2017). DNA methylation and obesity traits: An epigenome-wide association study. The REGICOR study. Epigenetics.

[23] Mendelson M.M., Marioni R.E., Joehanes R., Liu C.Y., Hedman A.K., Aslibekyan S., Demerath E.W., Guan W.H., Zhi D.H., Yao C. (2017). Association of Body Mass Index with DNA Methylation and Gene Expression in Blood Cells and Relations to Cardiometabolic Disease: A Mendelian Randomization Approach. PLoS Med..

[24] Richmond R.C., Sharp G.C., Ward M.E., Fraser A., Lyttleton O., McArdle W.L., Ring S.M., Gaunt T.R., Lawlor D.A., Smith G.D. (2016). DNA Methylation and BMI: Investigating Identified Methylation Sites at HIF3A in a Causal Framework. Diabetes.

[25] Selvaraj V., Stocco D.M. (2015). The changing landscape in translocator protein (TSPO) function. Trends Endocrin Met..

[26] Biswas L., Farhan F., Reilly J., Bartholomew C., Shu X. (2018). TSPO Ligands Promote Cholesterol Efflux and Suppress Oxidative Stress and Inflammation in Choroidal Endothelial Cells. Int. J. Mol. Sci..

[27] Giulietti M., Righetti A., Principato G., Piva F. (2018). LncRNA co-expression network analysis reveals novel biomarkers for pancreatic cancer. Carcinogenesis.

[28] Tan J.F., Jin X.F., Wang K.C. (2019). Integrated Bioinformatics Analysis of Potential Biomarkers for Prostate Cancer. Pathol. Oncol. Res..

[29] Tang Q.Q., Lane M.D. (1999). Activation and centromeric localization of CCAAT/enhancer-binding proteins during the mitotic clonal expansion of adipocyte differentiation. Genes Dev..

[30] Tang Q.Q., Lane M.D. (2012). Adipogenesis: From stem cell to adipocyte. Annu. Rev. Biochem..

[31] Guo L., Li X., Tang Q.Q. (2015). Transcriptional regulation of adipocyte differentiation: A central role for CCAAT/enhancer-binding protein (C/EBP) beta. J. Biol. Chem..

[32] Hastie N.D. (2017). Wilms’ tumour 1 (WT1) in development, homeostasis and disease. Development.

[33] Ndisang J.F., Tiwari S. (2014). Mechanisms by which heme oxygenase rescue renal dysfunction in obesity. Redox Biol..

[34] Lee K.Y., Luong Q., Sharma R., Dreyfuss J.M., Ussar S., Kahn C.R. (2019). Developmental and functional heterogeneity of white adipocytes within a single fat depot. EMBO J..

[35] Milagro F.I., Campion J., Cordero P., Goyenechea E., Gomez-Uriz A.M., Abete I., Zulet M.A., Martinez J.A. (2011). A dual epigenomic approach for the search of obesity biomarkers: DNA methylation in relation to diet-induced weight loss. FASEB J..

[36] Abete I., Gomez-Uriz A.M., Mansego M.L., De Arce A., Goyenechea E., Blazquez V., Martinez-Zabaleta M.T., Gonzalez-Muniesa P., De Munain A.L., Martinez J.A. (2015). Epigenetic Changes in the Methylation Patterns of KCNQ1 and WT1 after a Weight Loss Intervention Program in Obese Stroke Patients. Curr. Neurovasc. Res..

[37] Kang B.K., Moon T.W., Lee J.A., Park T.Y., Ko M.M., Lee M.S. (2012). The fundamental study for the standardisation and objectification of pattern identification in traditional Korean medicine for stroke (SOPI-Stroke): Development and interobserver agreement of the Korean standard pattern identification for stroke (K-SPI-Stroke) tool. Eur. J. Integr. Med..

[38] WHO Expert Consultation (2004). Appropriate body-mass index in Asian populations and its implications for policy and intervention strategies. Lancet.

[39] Jiang H.S., Lei R., Ding S.W., Zhu S.F. (2014). Skewer: A fast and accurate adapter trimmer for next-generation sequencing paired-end reads. BMC Bioinform..

[40] Messeguer X., Escudero R., Farre D., Nunez O., Martinez J., Alba M. (2002). PROMO: Detection of known transcription regulatory elements using species-tailored searches. Bioinformatics.

