# Supplementary material for: Hypermethylation of the TSPOAP1-AS1 Promoter May Be Associated with Obesity in Overweight/Obese Korean Subjects
Source: Int J Mol Sci. 2020 May 7;21(9):3307. doi: 10.3390/ijms21093307 (PMC7246878; doi:10.3390/ijms21093307)
Supplement: Supplementary file 1 [file ijms-21-03307-s001.pdf]

Supplemental table 1. Pilot study of whole genome bisulfate sequencing among individual normal subjects and overweight/obese subjects

TargetID	Position	Normal (N)				Overweight/obese (Ob)				N Mean	Ob Mean	Ob-N delta mean	*p* value
		N1	N2	N3	N4	Ob 1	Ob 2	Ob 3	Ob 4				
chr17-56402029	56402029	0.258	0.205	0.172	0.138	0.290	0.310	0.452	0.425	0.193	0.369	0.176	0.014
chr17-56402057	56402057	0.257	0.256	0.273	0.207	0.351	0.500	0.613	0.425	0.248	0.472	0.224	0.024
chr17-56402092	56402092	0.109	0.104	0.182	0.147	0.283	0.214	0.350	0.200	0.135	0.262	0.126	0.026
chr17-56402109	56402109	0.077	0.160	0.000	0.000	0.083	0.071	0.043	0.069	0.059	0.067	0.008	0.858
chr17-56402116	56402116	0.036	0.074	0.000	0.037	0.100	0.107	0.167	0.172	0.037	0.137	0.100	0.007
chr17-56402137	56402137	0.160	0.231	0.368	0.103	0.308	0.296	0.333	0.290	0.216	0.307	0.091	0.209
chr17-56402159	56402159	0.161	0.300	0.227	0.125	0.500	0.433	0.448	0.294	0.203	0.419	0.216	0.011
chr17-56402162	56402162	0.156	0.250	0.250	0.094	0.413	0.219	0.242	0.270	0.188	0.286	0.099	0.141
chr17-56402165	56402165	0.156	0.281	0.458	0.125	0.391	0.333	0.394	0.378	0.255	0.374	0.119	0.214
chr17-56402178	56402178	0.000	0.143	0.111	0.121	0.149	0.233	0.122	0.231	0.094	0.184	0.090	0.081
chr17-56402186	56402186	0.030	0.121	0.037	0.067	0.111	0.107	0.150	0.077	0.064	0.111	0.047	0.117
chr17-56402196	56402196	0.143	0.313	0.261	0.074	0.432	0.286	0.308	0.395	0.198	0.355	0.157	0.058
chr17-56402226	56402226	0.132	0.262	0.235	0.056	0.315	0.186	0.268	0.404	0.171	0.293	0.122	0.113
chr17-56402238	56402238	0.162	0.354	0.175	0.100	0.406	0.321	0.349	0.404	0.198	0.370	0.172	0.044

Supplemental table 2. Different DNA methylation level of CpG site in *TSPOAP1-AS1* promoter region between normal and obese patients

CpG No.	loci at chromosome	position from transcription start site	Normal (N=104)	overweight/obese (N=80)	*p*
CpG 1	56401981	-830	13.41±4.48[a]	14.97±5.52	0.027[b]
CpG 2	56401984	-827	18.72±5.33	19.96±6.47	0.102
CpG 3	56401992	-819	19.30±5.66	20.51±6.81	0.151
CpG 4	56401998	-813	12.04±4.38	13.19±4.90	0.045
CpG 5	56402029	-782	14.36±4.57	15.65±5.80	0.094
CpG 6	56402057	-754	23.49±6.55	24.95±7.73	0.098
CpG 7	56402092	-719	12.25±4.09	13.29±5.35	0.076
CpG 8	56402109	-702	5.29±2.55	5.91±3.26	0.09
CpG 9	56402116	-695	6.03±2.65	6.49±3.60	0.237
CpG 10	56402137	-674	15.43±4.63	16.54±5.69	0.061
**CpG 11**	**56402159**	**-625**	**23.73±6.19**	**25.84±7.29**	**0.033**
**CpG 12**	**56402162**	**-649**	**15.60±4.94**	**17.43±6.50**	**0.017**
**CpG 13**	**56402165**	**-646**	**20.57±5.29**	**22.82±7.01**	**0.010**
CpG 14	56402178	-633	8.81±3.75	9.84±4.17	0.09
CpG 15	56402186	-625	7.38±3.06	8.52±3.48	0.037
CpG 16	56402196	-615	20.48±5.74	22.27±6.45	0.047
CpG 17	56402226	-585	13.59±4.23	14.68±5.14	0.111
**CpG 18**	**56402238**	**-573**	**15.22±4.38**	**17.23±6.22**	**0.004**
**CpG 19**	**56402241**	**-570**	**19.59±5.36**	**22.22±6.89**	**0.003**
**CpG 20**	**56402247**	**-564**	**18.52±5.22**	**21.35±6.66**	**0.001**
CpG 21	56402253	-558	17.95±5.40	18.04±5.43	0.545
CpG 22	56402260	-551	24.66±6.45	26.18±6.90	0.051
CpG 23	56402269	-542	27.61±6.57	28.48±6.93	0.187
CpG 24	56402274	-537	24.18±6.55	25.85±7.15	0.048
CpG 25	56402276	-535	19.90±5.97	21.06±6.40	0.081
CpG 26	56402293	-518	18.10±5.99	19.61±6.04	0.041
CpG 27	56402305	-506	19.53±5.75	21.24±7.04	0.028
CpG 28	56402315	-496	19.86±6.16	21.38±7.14	0.064
CpG 29	56402317	-494	19.07±6.27	19.28±6.78	0.645
CpG 30	56402326	-485	18.24±6.18	19.11±6.98	0.169
CpG 31	56402330	-481	20.47±6.43	22.22±7.48	0.047
CpG 32	56402338	-473	18.77±6.61	19.44±6.66	0.388
CpG 33	56402342	-469	14.82±5.18	15.41±5.85	0.215
Mean			17.18±4.65	18.51±5.66	0.043

[a]: percent mean±SD of DNA methylation level. [b]: The *p* value calculated by binary general linear model after adjustment of sex, age, smoking and drinking.
